# Therapeutic potential of a novel peripherally restricted CB1R inverse agonist on the progression of diabetic nephropathy

**DOI:** 10.3389/fneph.2023.1138416

**Published:** 2023-03-28

**Authors:** Laetitia Jacquot, Océane Pointeau, Célia Roger-Villeboeuf, Patricia Passilly-Degrace, Rim Belkaid, Isaline Regazzoni, Julia Leemput, Chloé Buch, Laurent Demizieux, Bruno Vergès, Pascal Degrace, Glenn Crater, Tony Jourdan

**Affiliations:** ^1^ Pathophysiology of Dyslipidemia research group, National Institute of Health and Medical Research (INSERM) Unité Mixte de Recherche (UMR1231) Lipids, Nutrition, Cancer, Université de Bourgogne Franche-Comté, Dijon, France; ^2^ ImaFlow core facility, UMR1231 INSERM, University of Burgundy, Dijon, France; ^3^ Inversago Pharma Inc, Montréal, Québec, Canada

**Keywords:** nephropathy, diabetes, CB1 receptors, inverse agonism, peripherally-restricted, fibrosis

## Abstract

**Objective:**

This study assessed the efficacy of INV-202, a novel peripherally restricted cannabinoid type-1 receptor (CB1R) inverse agonist, in a streptozotocin-induced type-1 diabetes nephropathy mouse model.

**Methods:**

Diabetes was induced in 8-week-old C57BL6/J male mice *via* intraperitoneal injection of streptozotocin (45 mg/kg/day for 5 days); nondiabetic controls received citrate buffer. Diabetic mice were randomized to 3 groups based on blood glucose, polyuria, and albuminuria, and administered daily oral doses for 28-days of INV-202 at 0.3 or 3 mg/kg or vehicle.

**Results:**

INV-202 did not affect body weight but decreased kidney weight compared with the vehicle group. While polyuria was unaffected by INV-202 treatment, urinary urea (control 30.77 ± 14.93; vehicle 189.81 ± 31.49; INV-202 (0.3 mg/kg) 127.76 ± 20; INV-202 (3 mg/kg) 93.70 ± 24.97 mg/24h) and albumin (control 3.06 ± 0.38; vehicle 850.08 ± 170.50; INV-202 (0.3 mg/kg) 290.65 ± 88.70; INV-202 (3 mg/kg) 111.29 ± 33.47 µg/24h) excretion both decreased compared with vehicle-treated diabetic mice. Compared with the vehicle group, there was a significant improvement in the urinary albumin to creatinine ratio across INV-202 groups. Regardless of the dose, INV-202 significantly reduced angiotensin II excretion in diabetic mice. The treatment also decreased *Agtr1a* renal expression in a dose-dependent manner. Compared with nondiabetic controls, the glomerular filtration rate was increased in the vehicle group and significantly decreased by INV-202 at 3 mg/kg. While the vehicle group showed a significant loss in the mean number of podocytes per glomerulus, INV-202 treatment limited podocyte loss in a dose-dependent manner. Moreover, in both INV-202 groups, expression of genes coding for podocyte structural proteins nephrin (*Nphs1*), podocin (*Nphs2*), and podocalyxin (*Pdxl*) were restored to levels similar to nondiabetic controls. INV-202 partially limited the proximal tubular epithelial cell (PTEC) hyperplasia and normalized genetic markers for PTEC lesions. INV-202 also reduced expression of genes contributing to oxidative stress (*Nox2*, *Nox4*, and *P47phox*) and inflammation (*Tnf*). In addition, diabetes-induced renal fibrosis was significantly reduced by INV-202.

**Conclusions:**

INV-202 reduced glomerular injury, preserved podocyte structure and function, reduced injury to PTECs, and ultimately reduced renal fibrosis in a streptozotocin-induced diabetic nephropathy mouse model. These results suggest that INV-202 may represent a new therapeutic option in the treatment of diabetic kidney disease.

## 1 Introduction

Diabetic nephropathy (DN) is a complication of diabetes and a leading cause of chronic kidney disease (1). DN is defined as persistent macroalbuminuria associated with an alteration in creatinine clearance in the presence of diabetes. Two renal cell types are primarily affected: podocytes and proximal tubular epithelial cells (PTECs). Podocytes are epithelial cells located in the renal glomeruli that play a crucial role in maintaining glomerular selectivity, permeability, and protein filtration capacity (2, 3). Proteinuria is associated with morphological changes in the glomeruli causing dysfunction and podocyte detachment and/or apoptosis leading to failure of the glomeruli (4). The structural integrity of podocytes is central to kidney function and their failure purportedly plays a part in multiple renal diseases, including DN (5).

The PTECs, which are responsible for glucose reabsorption *via* expression of sodium-glucose co-transporters-2 (SGLT2), are also affected by the deleterious effects of diabetic chronic hyperglycemia. These cells commonly display tubular atrophy, apoptosis, and thickened tubular basement membranes with occasional splitting and lamination (6). In diabetes, expression of SGLT2 and glucose transporter 2 (GLUT2) is increased in PTECs, which in turn increases glucose re-absorption and contributes to hyperglycemia (7, 8). PTECs also express the multiligand receptor megalin, which is responsible for the normal proximal tubule uptake of filtered molecules, including carrier proteins, peptides, hormones and nephrotoxins. The expression of megalin, which plays an essential role in the development of some types of kidney injury, is significantly suppressed in diabetes (9, 10).

To slow progression of DN, blockade of the renin-angiotensin-aldosterone system (RAAS) *via* angiotensin II (Agt II) receptor antagonists or angiotensin converting enzyme (ACE) inhibitors is recommended (11, 12). With more advanced disease, the addition of SGLT2 transporter inhibitors is recommended for patients with type-2 diabetes (12) but not for type-1 diabetes because of an increased risk of ketoacidosis and acute kidney injury due to volume depletion (13). The use of SGLT2 transporter inhibitors (14) induces glycosuria by inhibiting glucose and sodium reabsorption in the PTECs, causing a mechanical reduction in blood glucose comparable to that observed with standard antidiabetic therapy, such as metformin. In addition, SGLT2 inhibitors confer a slight body weight loss, decrease systolic and diastolic blood pressure (15), and reduce the risk for nephropathy independent of their effect on blood glucose (16). Increasingly, patients require combination therapy targeting both the RAAS and SGLT2 transporter to control their diabetes, suggesting a need for new therapeutic targets.

Cannabinoid receptor blockers offer an interesting option for the treatment of DN. In the kidney, the cannabinoid receptor type 1 (CB1R) is mainly expressed in PTECs and podocytes, and plays an important role in the development of DN (17, 18). In animal models, CB1R inverse agonists improve function of these cells through reduced albuminuria, decreased glucose reabsorption, and improved glomerular filtration (19, 20). In addition to the direct effects on the kidney, CB1R inverse agonists have secondary reno-protective effects of weight loss, increased energy expenditure, and improved lipid profiles and glycemic control (21, 22).

Rimonabant, the first commercialized CB1R inverse agonist, yielded body weight loss and significant improvements in dyslipidemia, glycated hemoglobin, and glycemic control in obese and/or diabetic patients (23–25). However, rimonabant distributed to the brain and was removed from the market due to serious psychiatric adverse effects such as depression and suicidality (26). Alternatively, peripherally-restricted CB1R antagonists show at least equivalent efficacy to rimonabant in preclinical models, without the CNS penetrance underlying the central adverse effects of rimonabant (27). The objective of this study was to assess the efficacy of INV-202, a novel peripherally acting CB1R inverse agonist, originally described as MRI-1891 (28), in a type-1 diabetes DN mouse model.

## 2 Material and methods

### 2.1 Animals and disease induction

The study protocol was approved by the animal welfare ethics committee CE2A (APAFIS #16799 and 39296). Eight-week-old C57BL6/J male mice from Janvier Labs (Le Genest Saint Isle, France) were housed under a 12-hour light/dark cycle and fed a standard diet *ad libitum*. Diabetes was induced *via* intraperitoneal (IP) injection of streptozotocin (STZ; 45 mg/kg/day) in a sodium citrate solution (0.1 M, pH 4.5) for 5 consecutive days (n = 39). Nondiabetic control mice were administered citrate buffer IP for 5 days (n = 5).

Two weeks following STZ injection, mice with random glucose levels ≥ 230 mg/dL were considered diabetic (n = 24). Mice were kept for 12 more weeks for the nephropathy to fully develop, after which the mice were single-housed in metabolic cages (Techniplast, 3600M021, Decines-Charpieu, France) for 24 hours for urine collection. Diabetic mice were then divided into three groups (vehicle, INV-202 0.3 mg/kg and 3 mg/kg; n = 8 per group) with comparable blood glucose, polyuria, and albuminuria. During the pharmacological treatment period, 2 vehicle-treated and 1 INV-202 (0.3 mg/kg)-treated mice died, giving a final staff of 5 non-diabetic control, 6 vehicle-treated, 7 INV-202 (0.3 mg/kg) and 8 INV-202 (3 mg/kg) diabetic mice.

### 2.2 Pharmacological treatment and termination

Mice were administered daily oral doses for 28-days of either vehicle or INV-202 at 0.3 or 3 mg/kg (Kunos Laboratory, National Institute on Alcohol Abuse and Alcoholism, National Institutes of Health, Bethesda, USA). The formulation was DMSO/Tween 80/Saline (5/5/90). Previously, INV-202 exerted maximum efficacy in the diet induced obese mouse model at a dose of 3 mg/kg (which blocked 99.99% of CB1Rs in binding experiments) with only slight penetration across the blood-brain-barrier (28). The 10-fold lower dose of 0.3 mg/kg was included to further limit brain penetration while still blocking 90% of CB1Rs and to assess the dose relationship of INV-202 in the DN model (28).

On day 28, the mice were placed in metabolic cages for 24 hours for urine collection. Trunk blood was collected prior to euthanasia *via* a lethal injection of Euthasol followed by cervical dislocation. Kidneys were harvested, weighed, and snap frozen in liquid nitrogen or fixed in formalin for subsequent analysis. Given the significant body weight loss due to chronic diabetes, kidney weights were normalized using tibial length rather than body weight.

### 2.3 Biochemical markers

Albumin was measured in the urine pre- and post-treatment using an enzyme-linked immunosorbent assay (ELISA) kit (Mouse Albumin ELISA Kit; Bethyl Laboratories). Agt II was measured in urine and serum pre- and post-treatment using an ELISA kit (General Angiotensin II ELISA Kit; AbClonal Technologies; RK04203; Wuhan, China). Blood glucose was evaluated using a glucometer (MyLife Glucometer, Pura). Urinary urea, blood and urine creatinine were measured by the “Lipidomic Analytical Platform” of the University of Burgundy with a liquid chromatography with tandem mass spectrometry (LC-MS/MS) 1260 LC system coupled with a 6460 triple quadrupole MSMS detector (Agilent) equipped with a KINETEC column (2.6 µm HILIC 150 × 2.1 mm) at 30°C or 40°C, respectively. Urea was eluted at 0.2 mL/min in isocratic mode using methanol containing 0.1% formic acid. Creatinine was eluted at 0.3 mL/min in isocratic mode with a mixture of buffer A (acetonitrile/water, 95/5; ammonium acetate 5 mM) and buffer B (water, ammonium acetate 5 mM) in a 60/40 ratio. The analytes were quantitated using an isotopic dilution method with isotope-labeled standards—specifically, urea C13/15N2 (Santa Cruz Biotechnology; CAS 58069-83-3) or d3-creatinine (Santa Cruz Biotechnology; CAS 143827 20-7).

### 2.4 Histology

Experiments were carried out in the ImaFlow core facility, UMR1231 INSERM, University of Burgundy, Dijon, France. Kidneys were fixed in formalin (Labelians FPC60FT) for 48 hours at room temperature, dehydrated, and processed in paraffin (Leica ASP300 and Leica ASP300). Slices (5 µm) were prepared using a microtome (HistoCore Autocut R de Leica), then dried overnight at 37°C. Samples were stained with either hematoxylin and eosin (H&E), periodic acid-Schiff, or picro-sirius red, using an automated stainer (AutoStainer XL) after paraffin removal and sample rehydration.

Immunostaining with anti-Wilms Tumor 1 (WT-1; 1:400 in a 1% bovine serum albumin [BSA] solution) for 1 hour at room temperature (Nordic Biosite, ASC-9BJSZH) was performed manually on kidney slides. Slides were pretreated for 20 minutes with Tris-EDTA, pH 9 buffer at 95°C, inhibition of endogenous peroxidases for 15 minutes (3% hydrogen peroxide in 1X phosphate-buffered saline [PBS]), and saturation of nonspecific binding sites for 20 minutes before addition of primary antibodies (3% BSA + 2% milk powder in 1X TBS for WT-1 staining). For the anti-WT-1 staining it was necessary to block endogenous mouse Ig (1 hour at room temperature, Vector Laboratory kit MOM MP2400) before addition of the primary antibody. The signal was generated using a ​​​horseradish peroxidase (HRP)-labeled secondary antibody incubated for 30 minutes at room temperature and NovaRed substrate (Vector Laboratories SK4800). Counter-staining with Harris hematoxylin allowed for visualization of the nucleus (blue-violet). Slides were dehydrated and mounted in organic media. All images were acquired by optical microscopy (Axio Scope A1; Zeiss) coupled to Gryphax image acquisition software (Gryphax, Jenoptik, Jena, Germany). The different analyses were performed using Image J software (National Institutes of Health, Bethesda, USA).

### 2.5 Semi-Quantitative reverse transcription polymerase chain reaction

Kidney total RNA was extracted using the Tri Reagent technique, whereas total RNA from cells were extracted using a Qiagen (RNEasy^®^ Mini Kit) extraction kit. The concentration and purity of RNA was determined using a Nanophotometer N50 (Implen GmbH, Munich, Germany). Reverse transcription was done with 1 µg (*in vivo*) or 0.5 µg (*in vitro*) total RNA using an iScript Reverse Transcription Supermix for quantitative reverse transcription polymerase chain reaction (RT-qPCR; Bio-Rad Laboratories, #1708841). A Sybr Green Supermix kit (Bio-Rad Laboratories, #1708886) with QuantStudio 3 Real Time PCR System (ThermoFisher, Illkirch-Graffenstaden, France) were used for quantitative PCR. A standard curve was constructed for each gene using four dilutions of cDNA (1/5 to 1/100 dilution) and used to determine the relative variation in gene expression after normalization with the geometric mean of reference genes (*Hprt*, *Rpl19*, *Rpl32*, *Rplp0* and *Txn2)*. Primer sequences used for amplification are listed in **Table 1**.

**Table 1:** Primers sequence.

	Forward	Reverse
*Acta2*	CCCAGACATCAGGGAGTAATGG	TCTATCGGATACTTCAGCGTCA
*Agtr1a*	AACAGCTTGGTGGTGATCGTC	CATAGCGGTATAGACAGCCCA
*Ccl2*	TTAAAAACCTGGATCGGAACCAA	GCATTAGCTTCAGATTTACGGGT
*Cnr1*	CCGCAAAGATAGTCCCAATG	AACCCCACCCAGTTTGAAC
*Col1a1*	GCTCCTCTTAGGGGCCACT	CCACGTCTCACCATTGGGG
*Des*	GTTTCAGACTTGACTCAGGCAG	TCTCGCAGGTGTAGGACTGG
*Fn-1*	ATGTGGACCCCTCCTGATAGT	GCCCAGTGATTTCAGCAAAGG
*Hprt*	AGTCCCAGCGTCGTGATTAG	TTTCCAAATCCTCGGCATAATGA
*Lrp2*	AAAATGGAAACGGGGTGACTT	GGCTGCATACATTGGGTTTTCA
*Nox2*	CCTCTACCAAAACCATTCGGAG	CTGTCCACGTACAATTCGTTCA
*Nox4*	GAAGGGGTTAAACACCTCTGC	ATGCTCTGCTTAAACACAATCCT
*Nphs1*	GATGCGGAGTACGAGTGCC	GGGGAACTAGGACGGAGAGG
*Nphs2*	GACCAGAGGAAGGCATCAAGC	GCACAACCTTTATGCAGAACCAG
*P47phox*	ACACCTTCATTCGCCATATTGC	TCGGTGAATTTTCTGTAGACCAC
*Podxl*	AGGTGGTCAACCTTAATGGGG	TCATCCTTGGTCAAGTTGTCCA
*Rpl19*	ATGAGTATGCTCAGGCTACAGA	GCATTGGCGATTTCATTGGTC
*Rpl32*	TTAAGCGAAACTGGCGGAAAC	TTGTTGCTCCCATAACCGATG
*Rplp0*	AGATTCGGGATATGCTGTTGGC	TCGGGTCCTAGACCAGTGTTC
*Tgfb1*	CTCCCGTGGCTTCTAGTGC	GCCTTAGTTTGGACAGGATCTG
*Tim1*	ACATATCGTGGAATCACAACGAC	ACAAGCAGAAGATGGGCATTG
*Tnf*	CCCTCACACTCAGATCATCTTCT	GCTACGACGTGGGCTACAG
*Txn2*	TGGGCTTCCCTCACCTCTAAG	CCTGGACGTTAAAGGTCGTCA

### 2.6 Statistical analysis

Statistical analysis was performed with GraphPad Prism (version 7.04 for Windows, San Diego, CA) by an analysis of variance (ANOVA) followed by Tukey-Kramer *post-hoc* test for multiple comparisons. Time-dependent results were analyzed using an ANOVA followed by a Bonferroni test. Statistical significance was set at p < 0.05. All summary results are presented as mean ± standard error of the mean.

## 3 Results

### 3.1 Diabetes induction and randomization

**Figure 1:**
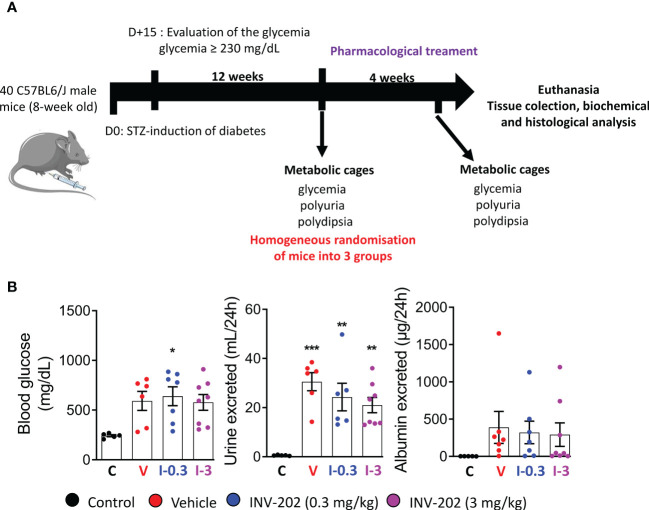
Induction of diabetes and randomization of diabetic mice. **(A)** Schematic of *in vivo* approach. **(B)** Randomization of mice with comparable glycemia, polyuria, and albuminuria into 3 groups: Control (n = 5), Vehicle (n = 6), INV-202 (0.3 mg/kg; n = 7), INV-202 (3 mg/kg; n = 8).Statistical significance versus nondiabetic controls: *p < 0.05; **p < 0.01; ***p < 0.001. STZ, streptozotocin. C: non-diabetic control mice, V: Vehicle-treated diabetic mice, I-0.3: INV-202-treated diabetic mice (0.3 mg/kg), I-3: INV-202-treated diabetic mice (3 mg/kg).

Of 40 mice initially, one mouse died prior to the start of the study, leaving 39 for randomization. Five were randomly selected as nondiabetic controls, while 34 were treated with STZ to induce diabetes. Only 24 mice had blood glucose levels ≥230 mg/dL and were evaluated for urine volume and albuminuria for randomization into 3 treatment groups (vehicle; INV-202 0.3 and 3 mg/kg; **Figure 1**).

### 3.2 INV-202 does not modify body weight nor hyperglycemia but can reverse renal hypertrophy and improve blood and urinary parameters

**Figure 2:**
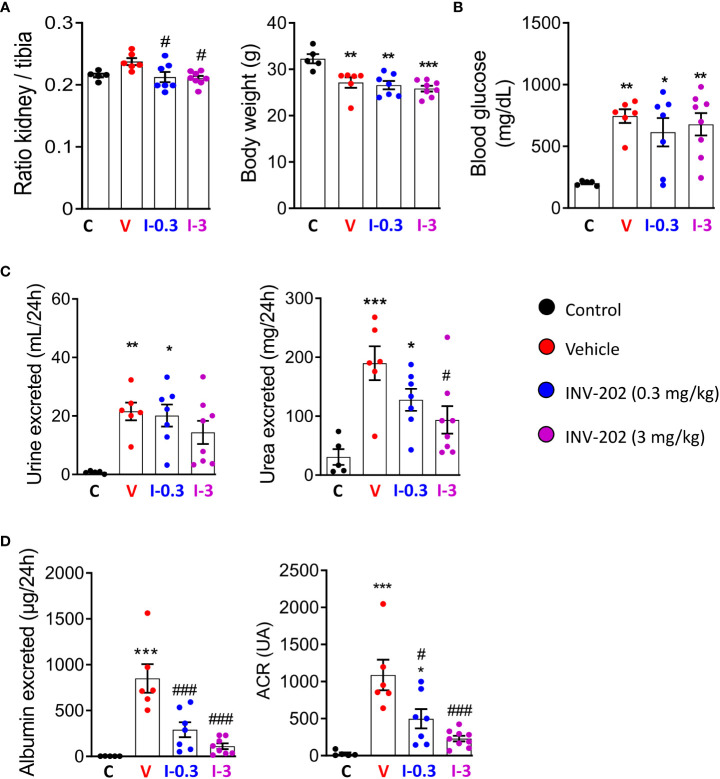
INV-202 treatment effects on different parameters associated with diabetic nephropathy. **(A)** Relative kidney mass and body weight following treatment. **(B)** Glycemia following treatment. **(C)** Polyuria and urinary urea following treatment. **(D)** Albumin excretion and urinary ACR following treatment. Statistical significance versus nondiabetic control: *p < 0.05; **p < 0.01; ***p < 0.001; versus vehicle: #p < 0.05; ###p < 0.001. ACR, albumin to creatinine ratio. C: non-diabetic control mice, V: Vehicle-treated diabetic mice, I-0.3: INV-202-treated diabetic mice (0.3 mg/kg), I-3: INV-202-treated diabetic mice (3 mg/kg).

Compared with non-diabetic mice, all STZ-induced diabetic mice displayed a similar weight loss that was unaffected by INV-202 (**Figure 2A**). However, INV-202 normalized the diabetes-induced renal hypertrophy after 28 days of treatment as compared with the vehicle group (**Figure 2A**). Also, no significant effect on glycemia was observed with INV-202 treatment, indicating that any effect of INV-202 on kidney function was mainly due to kidney remodeling rather than improved glycemic control (**Figure 2B**). While polyuria was unaffected by INV-202 treatment, urinary urea was significantly decreased versus vehicle in the INV-202 group at 3 mg/kg (**Figure 2C**). Compared with the vehicle group, there was a significant improvement in both INV-202 groups for albumin excretion and the urinary albumin to creatinine ratio (ACR; **Figure 2D**).

### 3.3 INV-202 decreased urinary excretion of Agt II and expression of Agt II and CB1R renal receptor

**Figure 3:**
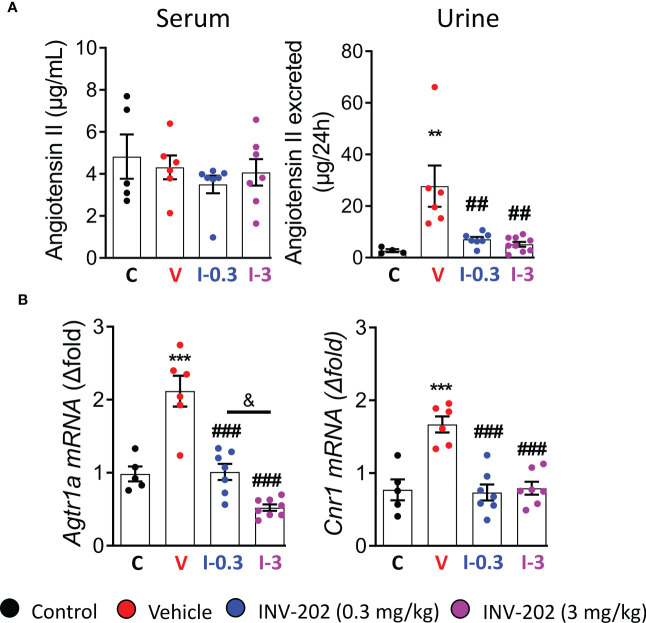
INV-202 treatment effects on angiotensin II excretion. **(A)** Serum and urinary angiotensin II concentration. **(B)** *Agtr1a* and *Cnr1* mRNA expression that codes for the angiotensin II receptor and the cannabinoid 1 receptor, respectively. Statistical significance versus nondiabetic control: **p < 0.01; ***p < 0.001; versus vehicle: ##p < 0.01; ###p < 0.001; versus INV-202 group, 0.3 mg/kg: & p < 0.05. C: non-diabetic control mice, V: Vehicle-treated diabetic mice, I-0.3: INV-202-treated diabetic mice (0.3 mg/kg), I-3: INV-202-treated diabetic mice (3 mg/kg).

The RAAS is highly involved in DN; its inhibition plays a significant role in the clinical treatment of the disease. Here, Agt II was quantified in the plasma and urine. Expression of *Agtr1a*, which codes for the Agt II receptor in the kidney, was also assessed. While plasma levels were similar across groups, the urinary Agt II excretion was increased in the vehicle group, and significantly reduced in the INV-202 groups regardless of dose (**Figure 3A**). Notably, a significant increase in *Agtr1a* and *Cnr1* (coding for CB1R) renal expression was seen in the vehicle group, which was significantly decreased in the INV-202 groups in a dose-dependent manner for *Agtr1a* while both doses normalized *Cnr1* expression (**Figure 3B**). This observation is of particular importance as CB1R may interact with Agt II signaling by heteromerization with its receptor in the kidney (29, 30).

### 3.4 INV-202 improves the glomerular filtration rate and prevents glomerular remodeling

**Figure 4:**
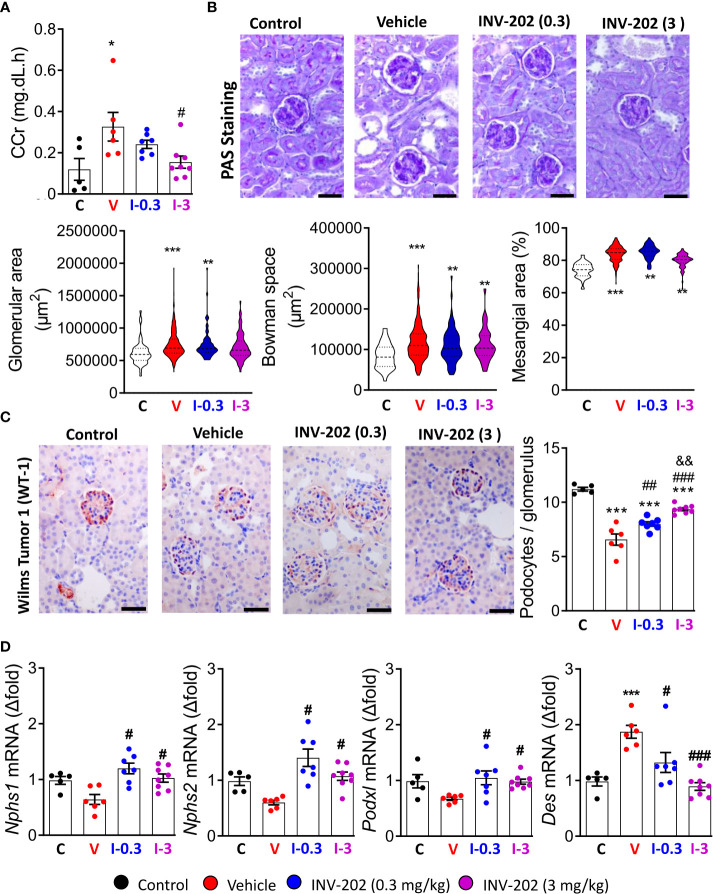
Effects of INV-202 treatment on glomeruli. **(A)** Summary of calculated CCrs. **(B)** Representative PAS staining performed on histological sections of kidneys from mice. Graphs summarize the mean glomerular area, Bowman space, and mesangial area (scale: 50 µm). **(C)** Representative immunostaining for Wilms tumor-1 protein (scale: 50 µm) with analysis of the mean podocyte numbers per glomerulus cross-section. **(D)** *Nphs1*, *Nphs2*, *Podxl*, and *Des* mRNA expression coding for nephrin, podocin, podocalyxin, and desmin, respectively. Statistical significance versus nondiabetic control: *p < 0.05; **p < 0.01; ***p < 0.001; versus vehicle: #p < 0.05; ##p < 0.01; ###p < 0.001; versus INV-202 group, 0.3 mg/kg: &p < 0.05; &&p < 0.01. CCr, creatinine clearance; PAS, periodic acid-Schiff. C: non-diabetic control mice, V: Vehicle-treated diabetic mice, I-0.3: INV-202-treated diabetic mice (0.3 mg/kg), I-3: INV-202-treated diabetic mice (3 mg/kg).

The glomerular filtration rate (GFR) is typically decreased in humans with chronic kidney disease. In our model, the pathology stage resembles the hyperfiltration observed in early or mid-impaired functions (31). We estimated GFR by assessing creatinine clearance (CCr). The mean CCr was increased in the vehicle group compared to control mice and significantly decreased in the INV-202 group at 3 mg/kg (**Figure 4A**). Such changes in kidney function are associated with glomerulopathy characterized by an increase in glomeruli size and in the mesangial space. These changes were observed in the diabetic vehicle group compared with nondiabetic controls but were reversed in the INV-202 groups in a dose-dependent manner (**Figure 4B**). Similarly, INV-202 preserved the Bowman space to a similar level as in nondiabetic mice (**Figure 4B**).

We quantified podocyte numbers per glomerulus section by immunostaining of the WT-1 protein, which is exclusively expressed in these cells in the kidney. The vehicle group showed a significant loss in the mean number of podocytes per glomerulus compared with nondiabetic controls, whereas the INV-202 groups showed limited podocyte loss in a dose-dependent manner (**Figure 4C**).

Gene expression of *Nphs1*, *Nphs2*, and *Pdxl*, which code for the podocyte structural proteins nephrin, podocin, and podocalyxin, respectively, was also assessed. Compared with nondiabetic controls, the vehicle group showed nonsignificant lowering of the expression of *Nphs1*, *Nphs2*, and *Podxl*, whereas expression of *Des* was significantly increased (**Figure 4D**). Desmin (*Des*) is a cytoskeletal protein with significant role in tissue proliferation and tubulointerstitial fibrosis in kidney (32). In both INV-202 groups, expression of these four markers were comparable to nondiabetic controls levels (**Figure 4D**).

### 3.5 INV-202 reduced renal lesions in PTECs

**Figure 5:**
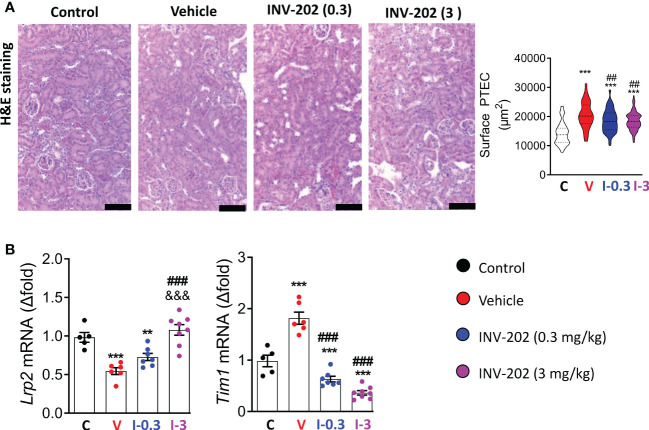
INV-202 treatment effects on proximal tubule cells. **(A)** Representative H&E staining of kidney slides with analysis of mean surface area of PTECs (scale: 100 µm). **(B)** mRNA analysis of *Lrp2* and *Tim1* coding for megalin and KIM1, respectively. Statistical significance versus nondiabetic control: **p < 0.01; ***p < 0.001; versus vehicle: ##p < 0.01; ###p < 0.001; versus INV-202 group, 0.3 mg/kg: &&&p < 0.001. H&E, hematoxylin and eosin; KIM1, kidney injury molecule 1; PTEC, proximal tubular epithelial cell. C: non-diabetic control mice, V: Vehicle-treated diabetic mice, I-0.3: INV-202-treated diabetic mice (0.3 mg/kg), I-3: INV-202-treated diabetic mice (3 mg/kg).

DN is often associated with a tubulopathy, characterized by lesions and hyperplasia of the PTECs (32). PTECs were identified on histological section with the help of the ImaFlow core facility. Hyperplasia of PTECs was found in the vehicle group and INV-202 groups compared with nondiabetic controls. INV-202 partially limited this hyperplasia (**Figure 5A**).

The genetic expression of megalin and of the kidney injury molecule 1 (KIM1) were measured. KIM1 is considered a useful marker of the presence of lesions in PTECs (33) and is involved in the detoxification mechanism of PTECs. The reduction of megalin expression (*Lrp2*) was greatest in the vehicle group compared with nondiabetic controls. Conversely, in the INV-202 group at 3 mg/kg, *Lrp2* expression did not significantly differ from nondiabetic controls (**Figure 5B**). Gene expression of kidney injury molecule 1 (*Tim1*) was significantly increased in the vehicle group versus nondiabetic controls and significantly decreased in both INV-202 groups relative to the vehicle and nondiabetic control groups (**Figure 5B**).

### 3.6 INV-202 reduced renal fibrosis

**Figure 6:**
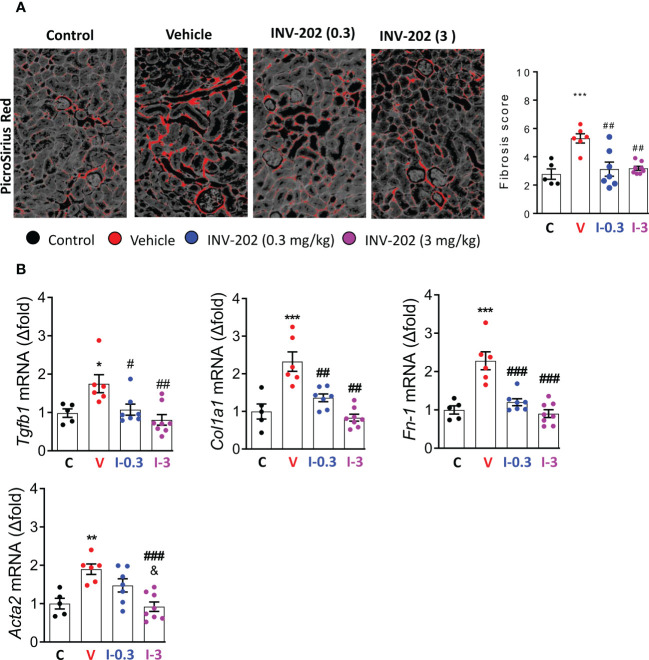
INV-202 reduces renal fibrosis. **(A)** Representative picro-sirius red stains of kidney slides for collagen fibers with analysis of mean fibrosis scores. **(B)** mRNA analysis of *Tgfb1, Col1a1, Fn-1* and *Acta2* coding for TGF-β, Collagen I type 1, fibronectin and α-smooth muscle actin respectively. Statistical significance versus the standard condition: *p < 0.05; **p < 0.01; ***p < 0.001; versus vehicle: #p < 0.05; ##p < 0.01; ###p < 0.001; versus INV-202 group. C: non-diabetic control mice, V: Vehicle-treated diabetic mice, I-0.3: INV-202-treated diabetic mice (0.3 mg/kg), I-3: INV-202-treated diabetic mice (3 mg/kg).

Compared with nondiabetic controls, renal fibrosis was significantly increased in the vehicle group. Both INV-202 groups exhibited a significant reduction in renal fibrosis compared with the vehicle group (**Figure 6A**), which was associated with a decrease in *Tgfb1* expression (**Figure 6B**). Moreover, Compared with control mice, the vehicle group displayed a marked increase in the gene expression of *Col1a1*, *Fn-1* and *Acta2*, coding respectively for Collagen, type I, alpha 1 fibronectin and α-smooth muscle actin (**Figure 6B**). Both INV-202 groups showed a significant reduction in the expression of those genes. This suggests that modulation of the transforming growth factor-beta (TGF-β) pathway contributed to the anti-fibrotic effect of INV-202.

### 3.7 INV-202 reduces gene expression for markers of renal oxidative stress and inflammation

**Figure 7:**
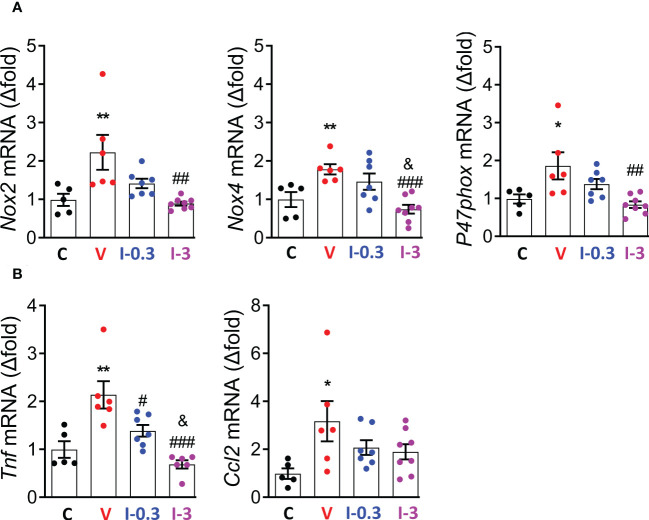
INV-202 reduces both renal oxidative stress and inflammatory markers. **(A)** mRNA analysis of *Nox2*, *Nox4*, and *P47phox* coding for NADPH oxidative isoforms (GP91Phox, NADPH oxidative 4, and neutrophil cytosol factor 1, respectively). **(B)** mRNA analysis of *Tnf* and *Ccl2* coding for TNF and C-C motif chemokine ligand 2. Statistical significance versus nondiabetic control: *p < 0.05; **p < 0.01; versus vehicle: #p < 0.05; ##p < 0.01; ###p < 0.001; versus INV-202 group, 0.3 mg/kg: &p < 0.05. NADPH, nicotinamide adenine dinucleotide phosphate; TNF, tumor necrosis factor. C: non-diabetic control mice, V: Vehicle-treated diabetic mice, I-0.3: INV-202-treated diabetic mice (0.3 mg/kg), I-3: INV-202-treated diabetic mice (3 mg/kg).

As the over-activation of the RAAS is associated with an upregulation of reactive oxygen species (ROS)-generating nicotinamide adenine dinucleotide phosphate (NADPH) oxidase isoforms in diabetic Zucker rats, we assessed the gene expression of GP91Phox (*Nox2*), NADPH oxidase 4 (*Nox4*), and *P47phox* (29). Compared with the vehicle group, expression of *Nox2*, *Nox4*, and *P47phox* was significantly reduced with INV-202 at 3 mg/kg (**Figure 7A**). Moreover, the expression of 2 inflammatory biomarkers was analyzed: tumor necrosis factor (*Tnf*) and chemokine ligand 2 (*Ccl2*). The expression of *Tnf*, but not *Ccl2*, was reduced with INV-202 in a dose-dependent manner (**Figure 7B**).

## 4 Discussion

Our STZ mouse model successfully reproduced various biological markers characteristic of human diabetes, including kidney hypertrophy, GFR alteration (estimated through the CCr alteration), and increased albuminuria. The latter marker is of particular clinical significance, as increased albumin excretion in urine (microalbuminuria) is an early sign of DN. Also, it is well established that kidney weight and GFR are affected from the onset of diabetes and progressively degenerate as DN develops (34). The significant reduction of CCr (predictive of GFR) with INV-202 treatment Compared with vehicle-treated mice was particularly noteworthy, as glomerular hyperfiltration is apparent early in the clinical course of diabetes and associated with an increased risk of progression to DN (35). However, while INV-202 restored these diabetic and DN markers, it did not restore polyuria or decrease glycemia and body weight, as reported previously in STZ mouse models treated with CB1R antagonists or displaying podocyte-specific genetic deletion of CB1R (19, 36). This suggests that the effects of INV-202 on renal parameters reported in this study are primarily due to remodeling of the kidney function, as opposed to an improvement of glycemic control. The latter is supported by similar findings that showed a disconnect between DN and hyperglycemia upon peripheral CB1R blockade, wherein DN improved while glycemic control remained unchanged (29).

DN is also associated with an over-activation of the RAAS and in particular of the activity of Agt II (37). Agt II triggers an increase in intra-glomerular pressure and stimulates the synthesis of growth factors such as TGF-β, which in turn promotes the accumulation of extracellular glomerular matrix and collagen expression, leading to the progressive formation of renal fibrosis (37). Inhibition of the RAAS is an important part of the treatment of DN. The CB1R can interact with Agt II and its receptor in the kidney (29). The latter represents a common pathway by which hyperglycemia and the RAAS elicits the various pathological changes involved in the development of DN. Thus, the decrease in Agt II urinary excretion and of its receptor expression *Agtr1a* observed *in vivo* may be attributable to INV-202’s CB1R inverse agonistic activity.

DN induces strong functional alterations in two cell types: the podocytes and the PTECs (4, 38). We observed an increase in the size of the glomeruli and of the mesangium in diabetic mice treated with vehicle. This is aligned with literature suggesting that DN is associated with significant structural changes in the glomeruli, such as glomerular hypertrophy, thickening of the basal membrane, and expansion of the mesangium (39, 40). These parameters were all improved following treatment with INV-202. Another important factor in the development of DN is the loss of podocyte pool, either *via* cell death or detachment and excretion in the urine (4) that leads to profound deleterious effects as the podocytes play a crucial role in maintaining glomerular selectivity and permeability, and in protein filtration (2, 3). Accordingly, we observed podocyte loss in the glomeruli of the diabetic vehicle-treated mice, which was reversed by INV-202 in a dose-dependent manner (WT-1 staining, **Figure 5**). We further demonstrated that INV-202 blocked the deleterious effects of diabetes on the expression of the genes (*Nphs1*, *Nphs2*, and *Pdxl*) coding for structural proteins (nephrin, podocin, and podocalyxin, respectively). This protection over podocyte loss is particularly important, as those cells are terminally differentiated and cannot replicate except in the presence of human immunodeficiency virus infection (41, 42).

DN is classically associated with an increase in oxidative stress and inflammation (43). In agreement with those features, the vehicle-treated diabetic mice displayed an increase in the expression of genes (*Nox2*, *Nox4*, and *P47phox*) coding for NADPH oxidative isoforms responsible for the generation of ROS and ensuing oxidative stress; and for the gene (*Tnf*) coding for TNF linked to inflammation. INV-202 mitigated those effects in a dose-dependent manner.

PTECs are the main site of glucose reabsorption *via* SGLT2 expression and activity (7). Diabetic nephropathy is often associated with tubulopathy, characterized by lesions and hyperplasia of the PTECs, and with cellular death triggered by a decrease in lipocalin-2 expression (44, 45). INV-202 treatment partially reduced tubular hyperplasia and blocked the negative effects of diabetes on lipocalin-2 protein expression. Moreover, INV-202 possibly improved PTEC function as gene expression of megalin was restored to normal levels. This is particularly relevant since megalin, which is typically decreased in diabetes, is both involved in the reabsorption process of proteins and in the detoxification process of the body (9, 10). Therefore, its inhibition during diabetes contributes to the development of albuminuria (46). We also observed a marked reduction in the expression of KIM1, a strong marker of PTEC lesions, which suggests a clear improvement in PTEC function and survival (33).

CB1R blockade has additional beneficial sodium-dependent and metabolic effects in PTECs. CB1R antagonism downregulated Na^+^/K^+^-ATPase activity in an *in vitro* PTEC (LLC-PK1) cell model (47) and in an ischemia and reperfusion LLC-PK1 kidney injury model (48). Accordingly, effects on CB1 inhibition may promote natriuresis, which would be beneficial in the management of hypertension, a major factor in the progression of diabetic kidney disease (49). Inhibition of Na^+^/K^+^-ATPase activity may further downregulate the sodium-dependent neutral amino acid transporter B0AT1, reducing amino acid bioavailability for the activation of mTORC1 (50). During hyperglycemia, blockade of CB1R also inhibits enhanced mTORC1 activity, downregulating GLUT2 expression in PTECs, which prevented diabetic mice from developing diabetic kidney disease (50).

Another interesting finding was the reduction in tubulo-interstitial fibrosis observed in INV-202 treated animals and associated reduction in TGF-β expression. Recently, CB1R was identified as a key player in the fibrogenic process in various tissues such as the liver (51, 52), lungs (53, 54), skin (55), and kidneys (56–58). In non-metabolic renal disease, CB1R gene expression is among the 10 most up-regulated genes in an experimental unilateral ureter obstruction model of renal fibrosis (56) and CB1R inhibition (genetic or pharmacological) profoundly reduces renal fibrosis (56), mainly through a direct action on renal interstitial myofibroblasts. Moreover, CB1R was described as a major contributor of chronic allograft dysfunction by inducing fibrosis (57). In all studies, reno-protection induced by CB1R modulation, either with neutral antagonists or inverse agonists, was associated with a reduction in TGF-β-mediated collagen deposition. The exact mechanism by which CB1R blockade reduces TGF-β signaling requires further study. However, it is reasonable to speculate that improvements in metabolic efficiency of the PTECs may allow them to better cope with oxidative stress, reducing senescence and other fibrotic pathways.

Lastly, improvements in several biomarkers were observed at the lowest dose of INV-202. Compared with vehicle, treatment with INV-202 0.3 mg/kg significantly decreased the mean excreted albumin, ACR, Agt II, gene expression of Agt II (*Agtr1a*) and CB1R (*Cnr1*) renal receptors, mesangial area, fibrosis score and *Tgfb1* gene expression, and expression of *Tnf* related to inflammation; and significantly increased the mean Bowman space, expression of genes coding for podocyte structural proteins (*Nphs1*, *Nphs2*, *Pdxl*), and preserved podocyte per glomerulus cross-section. This suggests that, even at low doses, INV-202 has the potential for beneficial effects on kidney function in patients with diabetic kidney disease.

In conclusion, we demonstrated the potential therapeutic effect of INV-202 in a STZ-induced DN mouse model. Our results suggest that INV-202 may represent a new therapeutic option in the treatment of diabetic kidney disease. Further studies in preclinical models are warranted to further clarify the mechanisms by which CB1R blockade confers protection. Clinical trials in humans using this therapeutic approach are also underway (NCT05514548).

## Data availability statement

The original contributions presented in the study are included in the article. Further inquiries can be directed to the corresponding author.

## Ethics statement

The animal study was reviewed and approved by CE2A, comité d’éthique de l’Université de Bourgogne (APAFIS #16799 and 39296).

## Author contributions

PD, BV, GC, and TJ conceived and planned the experiments. LJ, OP, CR-V, CB, IR, JL, PP-D, LD, and TJ carried out most of the experiments. RB performed histological staining and analysis. All authors provided critical feedback and helped shape the research, analysis, and manuscript. TJ is the guarantor of this work and, as such, had full access to all data in the study and takes responsibility for the integrity of the data and the accuracy of the data analyses. All authors contributed to the article and approved the submitted version.
